# Editorial: Skin-interfaced platforms for quantitative assessment in public health

**DOI:** 10.3389/fbioe.2024.1406483

**Published:** 2024-04-09

**Authors:** Seungju Han, Changhee Kim, Taehwan Kim, Hyoyoung Jeong, Sangmin Lee

**Affiliations:** ^1^ Department of Electronics and Information Convergence Engineering, Kyunghee University, Yongin, Republic of Korea; ^2^ Department of Electrical and Computer Engineering, University of California, Davis, Davis, CA, United States; ^3^ Department of Biomedical Engineering, Kyunghee University, Yongin, Republic of Korea

**Keywords:** skin-interfaced platforms, health monitoring, Bi-LSTM, blood pressure estimation, personalized healthcare

The development of wearable sensors for health monitoring applications has been greatly facilitated by advancements in soft electronics. Soft skin-interfaced electronics refer to electronic components and circuits that are designed to be flexible, stretchable, and gently interfaced with the human body. This design scheme enables comfortable and non-invasive integration with wearable devices, revolutionizing the field of public healthcare by providing numerous benefits in terms of comfort, wearability, and accuracy of quantitative health monitoring.

One of the main advantages of skin-interfaced electronics as wearable sensors is their flexibility and stretchability. Existing rigid electronics pose obstacles for skin-interfaced sensing applications as they are intrinsically uncomfortable to wear and may restrict movement. Skin-interfaced electronics, on the other hand, are fully integrated into flexible and thin substrates, enabling comfortable and unrestricted movement along with an improved signal-to-noise ratio. This flexibility ensures a better fit for the body, improving the accuracy and reliability of health monitoring data. In addition, such electronics are adaptable to biocompatible materials, so they are suitable for long-term wear on the skin without causing irritation or discomfort. This is particularly important for continuous health monitoring applications, where users may need to wear the device for a long period of assessment time. Soft materials, such as silicone elastomers and hydrogels, provide a comfortable interface between the sensor and the skin, ensuring user comfort and device adhesion.

Besides comfort and wearability, skin-interfaced electronics can offer high sensitivity and precision through signal processing, particularly in the quantitative assessment of public health applications. Soft sensors can be designed to detect a wide range of biometrics, including electrical, mechanical, optical, and even acoustic, such as cardiac activity, respiratory activity, body motion, body temperature, blood pressure, and biochemical markers in sweat or interstitial fluid. These sensor platforms can provide real-time monitoring of vital signs, allowing for early detection of health issues and timely intervention. Furthermore, soft electronics enable seamless integration of multiple sensors into a single device, offering comprehensive health monitoring capabilities. For example, a single device could integrate sensing elements for heart rate, respiratory rate, temperature, and activity level assessments, which can provide a holistic view of an individual’s health status. This engineered integration, combined with automated operation and machine learning-powered signal processing, significantly enhances usability, making daily health monitoring for individuals more natural and unobtrusive. As the field of wearable platform technology continues to advance, soft skin-interfaced electronics are expected to play an essential role in implementing new applications, especially in personalized remote healthcare monitoring.

Research in the field of skin-interfaced healthcare platforms has made significant advancements, widening the range of target sensing signals, quantizing collected data, and optimizing platforms. For instance, Tan et al., 2023 present research on measuring arterial and venous pulse at the wrist simultaneously, highlighting the importance of venous blood oxygen saturation monitoring. This establishes a foundation for noninvasive arterial and venous blood oxygen saturation measurement, and such signal processing techniques enhance the effectiveness of skin-interfaced electronics by allowing for enhanced accuracy and diverse biometric acquisitions (Tan et al., 2023). Jang et al. introduce a soft electronic system equipped with a 3D helical network structure for health monitoring that bypasses engineering constraints and performance limitations set by traditional 2D designs. The 3D interconnect buckling provides unique mechanical properties and enables physiological health monitoring wirelessly and without the need for batteries. This emphasizes the potential for advancements in medical applications as a methodological approach in the research of soft electronics substrates (Jang et al., 2017). Furthermore, Kwon et al., 2023 discuss the integration of flexible materials with conventional FPCB circuits, implementing soft packaging and miniaturization. This study explores methods of classifying signals obtained from portable sensors utilizing machine learning techniques, highlighting that these approaches can assist in assessments by portable monitoring, moving beyond traditional tethered clinical monitoring (Kwon et al., 2023).

## Conclusion

**FIGURE 1:**
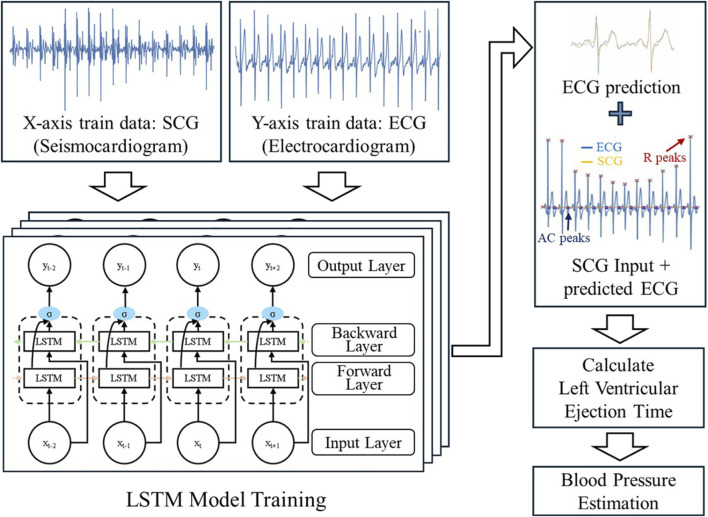
Illustration of the process for training a model utilizing a bidirectional Long short-term memory (LSTM) neural network to estimate blood pressure from electrocardiogram (ECG) and seismocardiogram (SCG) signals. The diagram at the top visualizes the network structure, including the input, bidirectional LSTM layers, and output layer. The results of the blood pressure estimation are displayed in the table on the bottom right.

Recent developments have primarily focused on miniaturization and packaging. As mentioned earlier, implementing soft packaging with multiple sensors enhances flexibility, comfort, and accuracy. Furthermore, advancements in AI models enable classification or discrimination of health signals obtained from personal skin-interfaced devices to extract more meaningful data. In this context, advanced AI models may yield valuable signals using reduced hardware resources. For example, blood pressure estimation using electrocardiogram (ECG) and seismocardiogram (SCG) can be explored. In particular, the study described in Figure 1 trained an AI model using a bidirectional Long short-term memory (LSTM) neural network to estimate blood pressure from ECG and SCG inputs. This approach allows for the prediction of ECG based on the collected SCG input. By inputting SCG into the trained model, the predicted ECG can be obtained. Subsequently, using the predicted ECG and measured SCG, it becomes possible to estimate blood pressure. Thus, using only the SCG sensor, it is possible to estimate ECG through an AI model, followed by blood pressure estimation based on the ECG and SCG signals relationship. This exemplified approach suggests a direction for development that leverages the aforementioned advantages of skin-interfaced devices and machine learning algorithms to enable more powerful quantitative assessments for public health applications.
